# Meal Frequency and Multi-Morbidity in a Cypriot Population: A Cross-Sectional Study

**DOI:** 10.3390/foods12183330

**Published:** 2023-09-05

**Authors:** Maria Kantilafti, Andria Hadjikou, Stavri Chrysostomou

**Affiliations:** 1 Department of Health Sciences, School of Sciences, European University Cyprus, Nicosia 2404, Cyprus; m.kantilafti@external.euc.ac.cy (M.K.); a.hadjikou@external.euc.ac.cy (A.H.); 2 Department of Life Sciences, School of Sciences, European University Cyprus, Nicosia 2404, Cyprus

**Keywords:** multi-morbidity, meal frequency, meal, snack

## Abstract

Data regarding the effect of specific dietary behaviors, such as meal frequency, on multi-morbidity are scarce. Therefore, the objective of this study was to examine the effect of meal frequency on multi-morbidity in a Cypriot population. A representative sample of 1255 adults >18 years old was surveyed during 2022–2023. Data regarding sociodemographic characteristics, multi-morbidity, and meal frequency consumption were collected through validated questionnaires. Diseases were listed according to the International Classification of Diseases, 10th Revision [ICD-10]. Statistical analysis was conducted using SPSS Statistics v.19.0. Responders who consumed more than three meals and snacks daily had a higher probability of multi-morbidity [OR: 1.505 [95% CI: 1.505–2.069]] compared with those who consumed three or fewer meals and snacks daily. The relation was not statistically significant after adjusting for age and gender and for socioeconomic characteristics. Furthermore, participants who consumed more than three snacks per day had a 1.776 [AOR: 1.616 [95% CI: 1.054–2.476]] higher risk of having multi-morbidity compared with participants who did not consume any snack or consumed one snack per day. The findings suggest that people with multi-morbidity have a higher risk when consuming three or more snacks per day regardless of age, gender, and socioeconomic characteristics.

## 1. Introduction

Eating occasion refers to the time that a person eats or drinks [1]. Meals are defined as specific eating occasions conducted between the hours of 6:00 and 10:00 a.m., 12:00 and 3:00 p.m., and 6:00 and 9:00 p.m. as breakfast, lunch, and dinner, respectively. Eating occasions at any other time are defined as snacks and they are characterized by the consumption of smaller quantities of food and terms of schedule [2,3]. However, based on the literature, there are several definitions of meals and snacks. Others define meals as any food consumed in the morning, noon, and night time which provides more than 150 kcals [4]. In regard to snacks, some definitions are based on the time of the day that the food is consumed [3,5,6], on the type of the food is consumed [7], on the amount of food consumed, or it can be a combination of the above. For example, based on [4], a snack is defined as food consumed at any time between meals and that provides 150 kcals maximum. Previous studies have documented that snacking helps people to achieve the daily recommended consumption of healthy foods, such as fruits, dairy products, dietary fibers, and other micronutrients like vitamins and minerals. Furthermore, snacking can help people to avoid digestive overload caused by fewer and larger meals [8,9]. On the other hand, other researchers reported that the consumption of high-energy-density snacks may contribute to obesity [10].

Several dietary habits, such as meal frequency, seem to have potential effects on human health and metabolism, especially on diet-related diseases [11,12]. Previous studies indicated that increased meal frequency is associated with a lower risk of obesity [13] and improved lipid profiles [14,15]. In regard to lipid metabolism, a previous meta-analysis of randomized controlled trials (RCTs) suggested that higher meal frequency improves lipid profile [16]. Moreover, other studies found that daily consumption of four meals compared with three meals was associated with a lower risk of obesity [5,17]. A meta-analysis of RCTs examining the effects of meal frequency on body weight found that increased meal frequency may beneficially affect body composition, indicating that meal frequency has a potential impact on diabetes prevention [18]. However, another recent meta-analysis of RCTs indicated that meal frequency may also affect body composition [19]. Also, the results of other studies investigating the relationship between meal frequency and type 2 diabetes (T2D) are conflicting. In particular, two studies did not find any association between meal frequency and the risk of T2D [20], while one study showed an increased risk of T2D in people consuming four meals per day compared with those consuming one to three meals per day [21]. However, three of the largest cohort studies in the US [Nurses’ Health Study; Health Professionals Follow-Up Study; Women’s Health Initiative Dietary Modification Trial] showed an increased risk of T2D among people having four or five meals per day [14].

Multi-morbidity is defined as the co-occurrence of two or more chronic diseases within a person [22,23], and it seems to affect a significant proportion of adults of all ages [24]. Based on the literature, the prevalence of multi-morbidity has a wide range and variation, between 10% and 90%, probably due to the use of different definitions, methods used for data collection, sociodemographic characteristics, and others [25]. However, the overall prevalence of multi-morbidity in adults has been estimated as 18 to 30% [24,26], and it is even higher in older people, varying between 55% and 98% [8]. Many factors have been shown to contribute to multi-morbidity, including sex, age, educational status, socio-economic status, levels of physical activity, smoking, sleep, and diet [27,28,29,30,31,32].

Regarding the relationship between diet and multi-morbidity, a recent study in Cyprus showed that higher adherence to the Mediterranean diet is associated with lower odds of multi-morbidity. However, more details about the potential effect of other factors, such as eating behavior, on multi-morbidity were not reported [32]. Indeed, it is well documented that a healthy diet, including high amounts of fruits and vegetables, whole grain products, fish, nuts, and low amounts of red meat and sweets, may decrease the risk of developing chronic metabolic diseases such as obesity, stroke, heart disease, hypertension, hyperlipidemia [33], and cancer [34]. However, data regarding the effect of specific dietary behaviors, such as meal frequency, on multi-morbidity are scarce. Therefore, the objective of this study was to examine the effect of meal frequency on multi-morbidity in a representative sample of the adult population in Cyprus.

## 2. Methods

### 2.1. Study Design

This was a cross-sectional study conducted from June 2022 to May 2023 within the five districts of the Republic of Cyprus (Famagusta, Larnaca, Limassol, Nicosia, and Paphos). The study was approved by the Cyprus National Bioethics Committee (EEΒK EP 2022.01.116). Moreover, all methods were carried out in accordance with relevant guidelines and regulations.

### 2.2. Study Population

Eligible participants for inclusion in the study were adults age > 18 years old with Cypriot citizenship who were permanently residing in Cyprus and willing to participate in the study. People living in hospitals, nursing homes, and rehabilitation centers were not eligible for participation.

### 2.3. Sample Size Determination

To ensure the representativeness of the sample, the sample was stratified according to the last demographic report (2011) by district, gender, and age. At first, the sample was divided into the five municipalities of Cyprus (Nicosia, Larnaca, Limassol, Famagusta, and Paphos). Afterwards, the sample was further divided in regard to sex and age creating four age groups (18–24, 25–44, 45–64, ≥65 years old). The sample’s national representativeness was confirmed using the chi-square goodness of fit test with statistical significance to be >0.05. Stratification ensures that the sample is representative of the population with respect to the chosen population parameters.

### 2.4. Data Collection

Data collection took place in public areas with a respond rate of 90%. The data were collected via a face-to-face interview using standardized questionnaires [35,36] and questionnaires used in previous studies which were conducted in Cyprus [32,37]. The questionnaires included questions about participants’ sociodemographic status, medical history, nutritional behaviors (e.g., meal and snack frequency consumption). Income of less than EUR 6500 per year was defined as “low-income” based on the Guaranteed Minimum Income (GMI) scheme in Cyprus [38]. Also, anthropometric data such as body weight (in kilograms) and height (in meters) were self-reported.

### 2.5. Assessment of Multi-Morbidity and Meal Frequency

Multi-morbidity is defined as the occurrence of two or more chronic conditions in the same individual [39]. Participants were asked if they have ever been diagnosed by a physician, with any of the questions included in the self-report validated questionnaire. The above-mentioned questionnaire consisted of forty-three chronic conditions according to the International Classification of Diseases, 10th Revision (ICD-10) including all basic human systems such as the circulatory, renal/urinary, reproductive, respiratory, skeletal/muscular, immune, respiratory, endocrine, digestive/excretory, and nervous systems. The above method used for the assessment of multi-morbidity for the same population had been used in a previous study [32]. For the assessment of meal frequency, self-reporting was also used. A meal was considered as any food consumed at breakfast, lunch, and dinner time [4]. In regard to snacks, snack was considered as any food consumed at any time between meals and providing less than 150 calories [4]. All participants were informed about the definitions of meals and snacks used in the current study. Participants were asked to report whether they consume one, two, three, or more than three meals and snacks during the day.

### 2.6. Statistical Analysis

In the descriptive statistics, the continuous variables (e.g., age and Body Mass Index (BMI)) are presented as mean ± standard deviation (SD), and categorical variables (e.g., age group, gender, BMI group, marital status, employment, income, and education) are presented as absolute and relative (%) frequencies. The baseline characteristics of participants according to multi-morbidity group were examined using Pearson’s chi-square test for categorical variables and the Mann–Whitney U test for continuous non-normally distributed variables. To determine the relationship between multi-morbidity and meal frequency consumption, three logistic regression models were conducted: first, multi-morbidity was regressed on meal frequency consumption as a crude model (model 1); second, the crude model was run, controlling for age and gender (model 2); and lastly, model 2 was further controlled for socioeconomic characteristics (marital status, occupational status, salary, educational status). For the logistic regression, the exposures were treated as categorical with more than two reference categories [reference category was the one with the lowest intake and the outcome (multi-morbidity) was modeled as dichotomous (presence vs. absence). Adjusted Odd Ratios (AORs) with 95% confidence intervals (95% CI) were reported. Statistical analysis was conducted using IBM SPSS Statistics v.29.0.

## 3. Results

Table 1 summarizes the physical and socioeconomic characteristics of the participants. A total of 1255 adults with mean age 43.43 ± 17.063 participated in the study. Results showed that age, BMI, marital status, employment status, income, and education were all significantly associated with multi-morbidity (*p* < 0.05). More specifically, it has been found that participants with multi-morbidity were older (57.926 ± 17.400 vs. 40.198 ± 15.213 years) and had a higher BMI (28.308 ± 5.940 vs. 25.313 ± 4.970 kg/m^2^) compared to individuals with none or one morbidity. Moreover, the prevalence of multi-morbidity was higher among separated, divorced, or widowed individuals (47.7%) compared with people who were married or living with a partner (20.0%) and unmarried people (6.5%). Furthermore, people with two or more chronic conditions, a lower salary (<EUR 6500), retirement, and lower education were more dominant factors compared to individuals with none or one chronic condition.

Table 2 presents the prevalence of multi-morbidity between individuals with different daily meal and snack consumption. It seems that participants with multi-morbidity consuming >3 meals and snacks daily are statistically more frequent compared to those with multi-morbidity consuming <3 meals and snacks during the day (*p* < 0.05). Moreover, the same is found for multi-morbidity and daily snack consumption. In particular, participants with multi-morbidity having ≥3 meals/day are significantly more frequent compared to those who have <3 meals per day (*p* < 0.05). Overall, it seems that the prevalence of multi-morbidity escalates when the number of daily snacks increases (*p* < 0.05).

Logistic regression analysis was conducted in order to examine the relation between multi-morbidity and daily meal and snack consumption (Table 3). Responders who consumed >3 meals and snacks daily had a higher probability of multi-morbidity (OR: 1.505 (95% CI: 1.505–2.069)) compared to those who consumed ≤3 meals and snacks daily. The relation was not statistically significant after adjusting for age and gender (model 2) and for socioeconomic characteristics (model 3).

Table 4 presents the results of the logistic regression investigating the association between multi-morbidity and daily snack consumption. Participants who consumed >3 snacks per day had a 1.776-fold (OR:1.776 (95% CI: 1.241–2.544)) higher risk of having multi-morbidity compared to participants who did not consume any snack or consumed 1 snack per day. The relation remained statistically significant after adjusting for possible confounders such as age and gender (model 2) (AOR:1.796 (95% CI: 1.199–2.689)) and socioeconomic characteristics (model 3) (AOR:1.544 (95% CI: 1.001–2.382)).

## 4. Discussion

To the best of our knowledge, this is the first study in Cyprus to examine the effect of meal frequency on multi-morbidity in a representative sample of the adult population. Our findings demonstrate that people with multi-morbidity were significantly older, with higher BMI compared to people without multi-morbidity. Moreover, separated/divorced and retired people with lower average yearly income (≤EUR 6500) and lower educational status (primary school) with multi-morbidity were significantly more compared to those without multi-morbidity. It is worth mentioning that a significant proportion of the Cypriot population has a yearly income lower than the minimum yearly wage in Cyprus, which is EUR 10,600 [38]. Also, regarding meal frequency, it seems that people with multi-morbidity consumed >3 meals and snacks during the day compared to people without multi-morbidity. Notably, the same was found for daily snack consumption. In particular, the risk of multi-morbidity increases when people consume ≥3 snacks per day regardless of age, gender, and socioeconomic characteristics. However, the frequency of main meal consumption was not different between people with multi-morbidity and people without multi-morbidity.

In relation to the association between socioeconomic characteristics and multi-morbidity, results from previous studies remain conflicting. A meta-analysis by Parathira et al., [40] which aimed to examine the association between socioeconomic status and multi-morbidity, showed that low education level was significantly associated with increased odds of multi-morbidity compared to a high educational level. In the same analysis, findings regarding the effect of income, sex, and age were controversial. However, similarly to our study, a recent study in China indicated that aging and BMI were positively associated with increased risk of multi-morbidity [41]. Similarly, other studies also found that the prevalence of multi-morbidity increased dramatically with aging and increased body weight [42,43,44,45]. A recent systematic review about the association between multi-morbidity, social conditions, and individual lifestyles demonstrated that low educational status is associated with a higher risk of multi-morbidity [46]. Authors also supported that people with a low level of educational status may have difficulties in finding and understanding health care information, and therefore, the risk of multi-morbidity is higher [46]. Concerning occupational status, previous studies showed that not working [47], being unemployed [48], or being pre-retired [49] increased the risk of having multi-morbidity. Similarly, our findings indicate that retired people with multi-morbidity were significantly more frequent compared to unemployed or employed people with multi-morbidity, and this could also be associated with aging. Therefore, based on our findings, morbidity is mediated by a population’s socioeconomic conditions.

Previous studies identified specific dietary patterns and food groups that are associated with the risk of morbidity. In particular, a study among 348,290 participants showed that the Western Pattern was associated with an increased risk of multi-morbidity, while the White Meat Pattern and the Prudent Pattern showed inverse associations. Moreover, for specific food groups, more frequent intakes of processed meat and poultry were associated with higher risks of multi-morbidity, whereas a higher intake frequency of fish and more intake of fruits and cereal were associated with decreased risks [50]. However, data regarding specific eating behaviors such as meal frequency and multi-morbidity are still not available.

The influence of meal frequency on health and disease has been a topic of interest for many years [11]. Notably, previous epidemiological data indicate an association between higher meal frequency and lower disease risk. In particular, studies reported reduced cardiovascular risk [51] and lower LDL cholesterol [52] with increased meal frequency. Also, a previous cohort study demonstrated that eating one to two times a day is associated with an increased risk of coronary heart disease compared to eating four to five meals per day [53]. Moreover, a previous study within the European Prospective Investigation into Cancer (EPIC) project showed that people reporting a higher (≥6 times/day) meal frequency had a lower concentration of total and LDL cholesterol, compared to those who ate one to two times a day [54]. In addition, a frequency of >4 times per day was associated with a lower risk of obesity compared to a frequency of <3 times per day [5], whereas, in another study, eating >6 meals per day reduces the risk of obesity compared to eating <3 meals daily [17]. Regarding diabetes, a sixteen-year follow-up study showed an increased risk of type 2 diabetes in men who ate one to two times a day compared to those who ate three meals a day [21]. Notably, most of the existing guidelines support that eating small and frequent meals is helpful for optimizing health in the general population. Indeed, the energy content of each snack and meal seems to be an important determinant.

In contrast to the beneficial effect of increased meal frequency on health, other studies seem to agree with our study, where meal frequency is positively associated with the risk of having two or more chronic diseases. As already mentioned earlier, previous studies investigated the effect of meal frequency on specific conditions and not on multi-morbidity. In particular, a recent study by [55] indicated a positive relationship between the number of meals and snacks (>3/daily) and BMI. In addition, recent prospective studies support that frequent snacking increases the risk of weight gain [56,57] and T2D [21,58]. Based on current and previous findings, increased meal frequency is associated with increased insulin secretion, increased food stimuli, hunger, and the desire to eat [11,59]. Thus, it could be assumed that higher meal frequency is associated with higher energy consumption leading to weight gain and other relative metabolic consequences.

Our findings regarding the effect of increased meal frequency on multi-morbidity seem to agree with the mechanisms of fasting dietary programs. There are several types of fasting programs. Indeed, in time-restricted fasting (TRF), feeding is eliminated within a defined time window, resulting in decreased meal frequency, inducing many health benefits compared to the normal daily meal distribution of 3–5 meals per day, spreading from breakfast to dinner [60]. Thus, many studies support that TRF is a new pattern with many potential effects on overall heath (diabetes, cancer, cardiovascular diseases, obesity). Similar to our study, snacking >3 times per day is also associated with multi-morbidity. In regard to snacking, researchers support that the daily inclusion of one to two snacks alleviates the potential metabolic overload caused by fewer heavier meals and might contribute to meeting recommendations for food groups (e.g., fruits, dairy) and nutrients like fiber and vitamins. However, it is very important to control the snack’s composition. Snacking needs to follow specific characteristics, mainly in terms of composition, in order to be optimal and beneficial for human health [8].

The strength of this study Is that, to the best of our knowledge, this is the first study to examine the effect of meal frequency on multi-morbidity in a representative study population. Potential limitations should be acknowledged; meal and snack composition, total energy intake (eating styles and macronutrient intake), and levels of physical activity are some major determinants influencing overall health status that were not considered as potential confounders. In addition, data collection was self-reported, increasing the probability of recall bias. Also, medical family history should be recognized in order to identify those cases with diseases characterized by a strong genetic predisposition that are less influenced by environmental factors. Moreover, the method used for data collection (self-reporting) could also lead to methodological bias.

## 5. Conclusions

In conclusion, our findings indicate that there is little robust evidence that reduced meal frequency is a beneficial strategy for reducing the risk of multi-morbidity. The currently available evidence is of low certainty and does not support current recommendations for reduced meal frequencies. The findings should be, however, interpreted with caution. Further studies are required to better elucidate the mechanisms through which meal frequency affects heath, disease occurrence, and, thus, multi-morbidity. Intervention studies, and particularly randomized controlled trials, should be performed on humans, including those who are healthy and those with multi-morbidity, in order to identify the optimal meal frequency for health maintenance and a reduced risk of multi-morbidity. Moreover, future studies should also include diet composition as a potential confounder together with meal frequency. Apart from diet composition, for the establishment of healthy eating patterns, it is important to consider eating behaviors including the optimal meal frequency.

## Figures and Tables

**Table 1:** Demographic and socio-economic characteristics overall and by multi-morbidity group.

	Total ^a^ (*n* = 1255)		Multi-Morbidity ^b^ (*n* = 229)		No Multi-Morbidity ^b^ (*n* = 1026)		*p* Value
Age (years)	43.43 ± 17.063		57.926 ± 17.400		40.198 ± 15.213		**<0.001 ^c^**
Age group							**<0.001 ^d^**
18–24	194	(15.5)	9	(4.6)	185	(95.4)	
25–44	507	(40.4)	44	(8.7)	463	(91.3)	
45–64	357	(28.4)	76	(21.3)	281	(78.7)	
≥65	197	(15.7)	100	(50.8)	97	(49.2)	
Gender							0.81 ^d^
Male	572	(45.6)	92	(16.1)	480	(83.9)	
Female	683	(54.4)	137	(20.1)	546	(79.9)	
BMI (kg/m^2^)	25.859 ± 5.2864		28.308 ± 5.940		25.313 ± 4.970		**<0.001 ^c^**
BMI group (kg/m^2^)							**<0.001 ^d^**
Underweight (<18.5)	40	(3.2)	3	(7.5)	37	(92.5)	
Normal weight (18.5–24.9)	577	(46)	73	(12.7)	504	(87.3)	
Overweight (25–29.9)	424	(33.8)	82	(19.3)	342	(80.7)	
Obese (≥30)	214	(17.1)	71	(33.2)	143	(66.8)	
Marital status							**<0.001 ^d^**
Married or living with partner	825	(65.7)	165	(20.0)	660	(80.0)	
Never married	339	(27.0)	22	(6.5)	317	(93.5)	
Separated, divorced or Widowed	86	(6.9)	41	(47.7)	45	(52.3)	
Employment							**<0.001 ^d^**
Missing 4 (0.3)							
Employed	883	(70.6)	119	(13.5)	764	(86.5)	
Unemployed	198	(15.8)	21	(10.6)	177	(89.4)	
Retired	170	(13.6)	89	(52.4)	81	(47.6)	
Income (yearly average)							**<0.001 ^d^**
Missing 88 (7.0)							
≤EUR 6500	206	(17.7)	63	(30.6)	143	(69.4)	
EUR 6500–EUR 19,500	517	(44.3)	90	(17.4)	428	(82.6)	
≥EUR 19,500	444	(38.0)	71	(16.0)	373	(84.0)	
Education							**<0.001 ^d^**
Missing 5 (0.4)							
Primary	77	(6.2)	48	(62.3)	29	(37.7)	
Secondary	314	(25.1)	74	(23.6)	240	(76.4)	
Higher education	859	(68.4)	106	(12.3)	753	(87.7)	
Abbreviations: BMI: Body Mass Index							

Bold values represent statistically significant associations (*p* < 0.001). ^a^ Means ± sd. ^b^ N (%). ^c^ Mann–Whitney U test. ^d^ Pearson’s chi-squared test.

**Table 2:** Prevalence of multi-morbidity by meal and snack frequency consumption.

	Total (*n* = 1255)		Multi-Morbidity (*n* = 229)		No Multi-Morbidity (*n* =1026)		*p* Value
Main meals and snacks (daily)							**0.014**
Missing 5 (0.4)							
1–3	428	(34.2)	62	(14.5)	366	(85.5)	
>3	822	(65.8)	167	(20.3)	655	(79.7)	
Main meals (daily)							0.648
Missing 2 (0.2)							
1–2	523	(41.7)	92	(17.6)	431	(82.4)	
≥3	730	(58.3)	137	(18.8)	593	(81.2)	
Snacks (daily)							**0.003**
Missing 5 (0.4)							
0–1	500	(40.0)	79	(15.8)	421	(84.2)	
2	462	(37.0)	76	(16.5)	386	(83.5)	
≥3	288	(23.0)	72	(25.0)	216	(75.0)	

Bold values represent statistically significant associations (*p* < 0.05).

**Table 3:** Logistic regression analysis to evaluate the association of daily meal and snacks consumption with multi-morbidity presence.

	Total (*n* = 1250)	
	OR	95% CI
Model 1: Crude model		
Meals and snacks daily frequency (1–3/>3)	**1.505**	**1.095–2.069**
Model 2: Model 1 adjusted for age and gender		
Meals and snacks daily frequency (1–3/>3)	1.388	0.976–1.975
Model 3: Model 2 plus adjusted for socioeconomic characteristics ^a^		
Meals and snacks daily frequency (1–3/>3)	1.132	0.771–1.662

^a^ Marital status, occupational status, salary, educational status, BMI. OR and their corresponding 95% CI were obtained from binary logistic regression analysis. Bold values represent statistically significant associations.

**Table 4:** Logistic regression analysis to evaluate the association of daily snacks consumption with multi-morbidity presence.

	Total (*n* = 1250)	
	OR	95% CI
Model 1: Crude model		
Snacks daily frequency		
0–1	Ref.	
2	1.049	0.744–1.480
≥3	**1.776**	**1.241–2.544**
Model 2: Model 1 adjusted for age and gender		
Snacks daily frequency		
0–1	Ref.	
2	1.039	0.707–1.525
≥3	**1.796**	**1.199–2.689**
Model 3: Model 2 plus adjusted for socioeconomic characteristics ^a^		
Snacks daily frequency		
0–1	Ref.	
2	0.971	0.646–1.460
≥3	**1.544**	**1.001–2.382**

^a^ Marital status, occupational status, salary, educational status, BMI. OR and their corresponding 95% CI were obtained from binary logistic regression analysis. Bold values represent statistically significant associations.

## Data Availability

The data used to support the findings of this study can be made available by the corresponding author upon request.

## Abbreviations

AOR—adjusted odds ratio; CI—confidence interval; RCTs—randomized controlled trials; T2D—type 2 diabetes; BMI—body mass index; EPIC—European Prospective Investigation into Cancer; TRF—time-restricted fasting.
